# Influencing the secretion of myogenic factors from mesenchymal stem cells

**DOI:** 10.1186/scrt485

**Published:** 2014-08-13

**Authors:** Lucas R Smith

**Affiliations:** Department of Anatomy and Cell Biology, School of Dental Medicine, University of Pennsylvania, 2040 S. 40th Street, Philadelphia, PA 19104 USA

## Abstract

Mounting evidence indicates that the regenerative effect of mesenchymal stem cells in skeletal muscle is related to the secretion of factors that stimulate resident myogenic cells. However, the environmental cues that affect the secreted factors of mesenchymal stem cells are not well understood. A recent publication demonstrated that secretion of factors is dependent on cell substrate, with mesenchymal stem cells grown on laminin providing more pro-myogenic factors than those grown on collagen, and that cellular strain may also play a role. Conditioned media from mesenchymal stem cells grown on laminin and subjected to strain provided the quickest and largest stimulation to myogenic cell proliferation. The influence of cell substrate and mechanical perturbation on mesenchymal stem cells therefore appears key to secretion of factors that support myogenesis.

## Introduction

Skeletal muscle’s robust ability to regenerate is compromised in a myriad of diseases, ranging from genetic muscular dystrophies to sarcopenia, with few therapeutic options available for those suffering from these conditions. One commonality among these conditions is an alteration in the local muscle environment, particularly prolific extracellular matrix components and fibrosis. The primary cell responsible for regeneration of skeletal muscle is the tissue-resident satellite cell; however, the *in vivo* cues that lead to satellite cell-based regeneration are not completely understood. Mesenchymal stem/stromal cells (MSCs) are increasingly being viewed as an instigator for satellite cell activation and coordinator of muscle regeneration [1, 2]. De Lisio and colleagues [1] have recently investigated how the extracellular matrix and mechanical environment of MSCs refines their ability to promote myogenesis.

MSCs play a role in homeostasis and regeneration in many tissues, and they have been shown to inhibit degeneration and enhance regeneration in a variety of tissues, including skeletal muscle [3]. MSCs present an exciting potential for cell-based therapy due to their ease of isolation and immune privileged status and their vigorous ability to expand in culture. MSCs may support tissue regeneration through direct differentiation due to their multi-lineage potential, but more likely act via secretion of factors that stimulate native tissue repair processes [4]. Within skeletal muscle the variety of supportive mesenchymal progenitor cells has been an active area of research [2]. While the possibility that these cells become myogenic remains, their potential to regulate muscle regeneration through secretion of pro-myogenic factors is clear.

What is less clear is how the MSC secrotome responds to a diseased environment. In skeletal muscle the diseased environment is often associated with fibrosis, with MSCs exposed to an abundance of collagen. Skeletal muscle is a highly dynamic tissue in which the local environment includes frequent large strains that have the potential to manipulate the MSCs as well. A study by De Lisio and colleagues [1] in the previous issue of *Stem Cell Research & Therapy* investigated the effect of the strain and substrate on the ability of MSCs to promote myogenesis. The authors isolated MSCs resident in skeletal muscle of transgenic mice overexpressing α7 integrin, providing greater cell yields compared to wild-type mice [5]. The MSCs were cultured on either laminin or collagen I and subsets of each were subjected to a 5 hour strain protocol. Neither substrate nor strain had an impact on the proliferation of the MSCs. While none of the MSCs differentiated toward a muscle fate, those grown on laminin did express increased levels of myogenic factors (Myf5, MyoD, and myogenin). Additionally, growth and inflammatory factor expression by MSCs was reduced on collagen substrates, which mimic the fibrotic muscle environment, including vascular endothelial growth factor A, interleukin-6, and NF-κB. Surprisingly, transforming growth factor β1 was also decreased on the collagen substrate despite its hypothesized role as a key component of the positive feedback loop leading to progressive muscle fibrosis [6]. The collagen substrate also led to a decrease in laminin expression; however, the expression of collagen was not affected by substrate. The authors hypothesize that these changes in expression may be mediated through activation of focal adhesion kinase (FAK), a known player in mechanotransduction [7]. While p-FAK did increase on stretched laminin substrate, this result was not observed in myogenic factor expression, implicating other methods for signal transduction driving MSCs to secrete myogenic factors. This investigation lays a foundation to test other factors secreted by MSCs that promote muscle regeneration, such as matrix metalloproteinases [8], for their dependence on substrate and strain.

Undoubtedly the milieu of factors being secreted by supportive cells, including MSCs, in skeletal muscle is complex. While the study of which individual secreted factors play a role progresses, assessing the gamut of factors is necessary to delimit the potential of environmental factors in MSCs in support of myogenesis. By applying conditioned media from MSCs grown in the environments outlined previously to myoblasts, a range of factors and their interactions have been observed. MSCs grown on laminin and subjected to stretch produced media that 24 hours after transfer to myoblast culture increased the proliferation of myoblasts compared to conditioned media from other MSC environments. This result fits in well with the activation of FAK observed in MSCs on stretched laminin. However, after 48 hours the number of myoblasts was greatest when exposed to media from MSCs grown on collagen. The inconsistent effect on proliferation with time confuses definition of the optimal substrate for MSCs to support myoblast proliferation long term. Myoblast proliferation is seen as the major factor altered by MSC conditioned media as signs of hypertrophy in myotubes were not present.

De Lisio and colleagues have provided strong evidence that the secretome of MSCs is influenced by the substrate they are attached to and may also be regulated by strain. The alteration of secreted factors by substrate and strain has effects on myoblast proliferation, but additional work is required to optimize the environment in which MSCs support myogenesis. There are also indirect avenues for the secretome of MSCs to exert influence on myoblasts, as evidenced by strain inducing MSCs to produce factors promoting vessel remodeling [9]. De Lisio and colleagues have just cracked the door open on the interaction between the muscle MSC environment and their secretion of pro-myogenic factors, as a multitude of further interactions are possible. For example, insulin-like growth factor 1 has been shown to be a potent activator of myogenesis [10] and enhance repair when overexpressed by MSCs [11]. Eventually these lessons learned in culture will need to be transferred to an *in vivo* system. A key issue in the development of MSCs for therapeutic purposes is how quickly the MSCs respond to these environmental cues. Will pre-conditioned MSCs maintain their pro-myogenic secretions once injected into a diseased muscle environment? Continued efforts such as these to study the effect of local environments on the multitude of cells in skeletal muscle will be necessary to tailor treatments to diseased muscle.

## Abbreviations

FAK: Focal adhesion kinase
MSC: Mesenchymal stem/stromal cell.
